# Genome-Wide Analysis of Serine/Arginine-Rich Protein Family in Wheat and *Brachypodium distachyon*

**DOI:** 10.3390/plants8070188

**Published:** 2019-06-26

**Authors:** Shoukun Chen, Jiawei Li, Yue Liu, Haifeng Li

**Affiliations:** State Key Laboratory of Crop Stress Biology for Arid Areas, College of Agronomy, Northwest A & F University, Yangling 712100, China

**Keywords:** *Brachypodium distachyon*, wheat, serine/arginine-rich (SR) protein, alternative splicing

## Abstract

By regulating the pre-mRNA splicing of other genes and themselves, plant serine/arginine-rich (SR) proteins play important roles in development and in response to abiotic stresses. Presently, the functions of most plant SR protein genes remain unclear. Wheat (*Triticum* *aestivum*) and *Brachypodium* *distachyon* are closely related species. In this study, 40 TaSR and 18 BdSR proteins were identified respectively, and they were classified into seven subfamilies: SR, RS, SCL, RSZ, RS2Z, SC35, and SR45. Similar to *Arabidopsis* and rice SR protein genes, most TaSR and BdSR protein genes are expressed extensively. Surprisingly, real-time polymerase chain reaction (RT-PCR) analyses showed that no alternative splicing event was found in TaSR protein genes, and only six BdSR protein genes are alternatively spliced genes. The detected alternatively spliced BdSR protein genes and transcripts are much fewer than in *Arabidopsis*, rice, maize, and sorghum. In the promoter regions, 92 development-related, stress-related, and hormone-related *cis*-elements were detected, indicating their functions in development and in response to environmental stresses. Meanwhile, 19 TaSR and 16 BdSR proteins were predicted to interact with other SR proteins or non-SR proteins, implying that they are involved in other functions in addition to modulating pre-mRNA splicing as essential components of the spliceosome. These results lay a foundation for further analyses of these genes.

## 1. Introduction

Pre-mRNA splicing is an important mechanism to regulate gene expression in eukaryotes, and this mechanism makes alternative splicing (AS) possible. Pre-mRNAs of eukaryotes are spliced by the spliceosome, a large complex containing five small nuclear ribonucleo-proteins (snRNPs) and non-snRNP proteins [1]. Serine/arginine-rich (SR) proteins, one kind of the non-snRNPs, were originally identified as factors necessary for the splicing of pre-mRNA in metazoans [2]. SR proteins are characterized by one or two RNA recognition motifs (RRMs) in the N-terminus and one arg/ser-rich (SR) domain in the C-terminus [3]. The RRM motifs recognize and bind to a variety of mRNA regulatory elements [3]; whereas the RS domain is required for protein-protein interactions [4].

Plant SR proteins were first identified in *Arabidopsis* [5,6]. There are 18 and 20 SR protein genes in *Arabidopsis* and rice (*Oryza sativa*) respectively [7,8,9]; and there are 21 maize (*Zay mays*) and 18 sorghum (*Sorghum bicolor*) SR protein genes [10]. Plant SR proteins could be classified into seven subfamilies: SR, RS, SCL, RSZ, RS2Z, SC35, and SR45 (SR-like). Among them, RS, RS2Z, and SCL are plant-specific [11]. RS members have two RRM domains, but the second RRM domain lacks the SWQDLKD motif, which is a characteristic of members in the SR subfamily. RS2Z subfamily member has two Zn-knuckles and an SP-rich region [12].

As components of the spliceosome, SR proteins regulate constitutive and alternative splicing. Overexpression of *AtSRp30*, resulted in changed AS of several genes, including *AtSRp30* itself. Consequently, the development of transgenic plants is delayed [13]. Similarly, overexpression of *AtRSZ33* showed its function in pre-mRNA splicing, and resulted in abnormal development [14]. By generating and analyzing multiple mutants of *SC35* and *SCL* genes, Yan et al. found that the splicing patterns of many genes were affected, and the mutant plants displayed pleiotropic changes in morphology and development [15]. Similarly, rice *RSp29* and *RSZp23* function in pre-mRNA splicing [7].

SR protein genes are extensively alternatively spliced genes. In *Arabidopsis*, 13 SR protein genes are alternatively spliced and generate 75 transcripts at the seedling stage, and 53 contain premature termination codons (PTC) [16]. When treated with hormones or under abiotic stresses, 15 *Arabidopsis* SR protein genes produce 95 transcripts [17]. In rice, at least 8 SR transcripts generate more than one isoform [7]. In maize and sorghum, 92 and 62 SR transcript isoforms were detected respectively [10]. However, the detailed functions of most plant SR protein genes and the significance of the abundant AS events remain unclear.

Probably, one exception is *Arabidopsis* SR45 [18]. As mentioned above, overexpression of Arabidopsis SRp30, RSZ33, or mutations in SC35 and SCL genes cause abnormal development [13,14,15]. In addition, SR proteins might be involved in response to stresses. When treated with ABA (abscisic acid), the expression of SCL30a, SCL28, and SCL33 are changed [19]. Cold, heat, and hormones dramatically alter the AS of several *Arabidopsis* SR protein genes, indicating the involvement in response to environmental stresses [17]. *Arabidopsis SR34b* participates in the resistance to cadmium [20]. In addition to response to high light [21], *Arabidopsis SR45* acts as a suppressor of innate immunity [22]. *Arachis diogoi* SR protein RSZ21 plays certain roles in plant defense and HR-like cell death [23]. These results indicated that SR proteins are involved in response to environmental stresses.

Belonging to pooideae subfamily, wheat and *B. distachyon* are close species. The former is a main cereal crop [24], and the latter is a new model plant of grasses. Here, we conducted a comprehensive analysis of wheat and *B. distachyon* SR protein genes at the genome-wide scale. This study lays a foundation for further functional elucidation of these genes.

## 2. Results

### 2.1. Serine/Arginine-Rich (SR) Proteins in Wheat and B. distachyon

**Figure 1:**
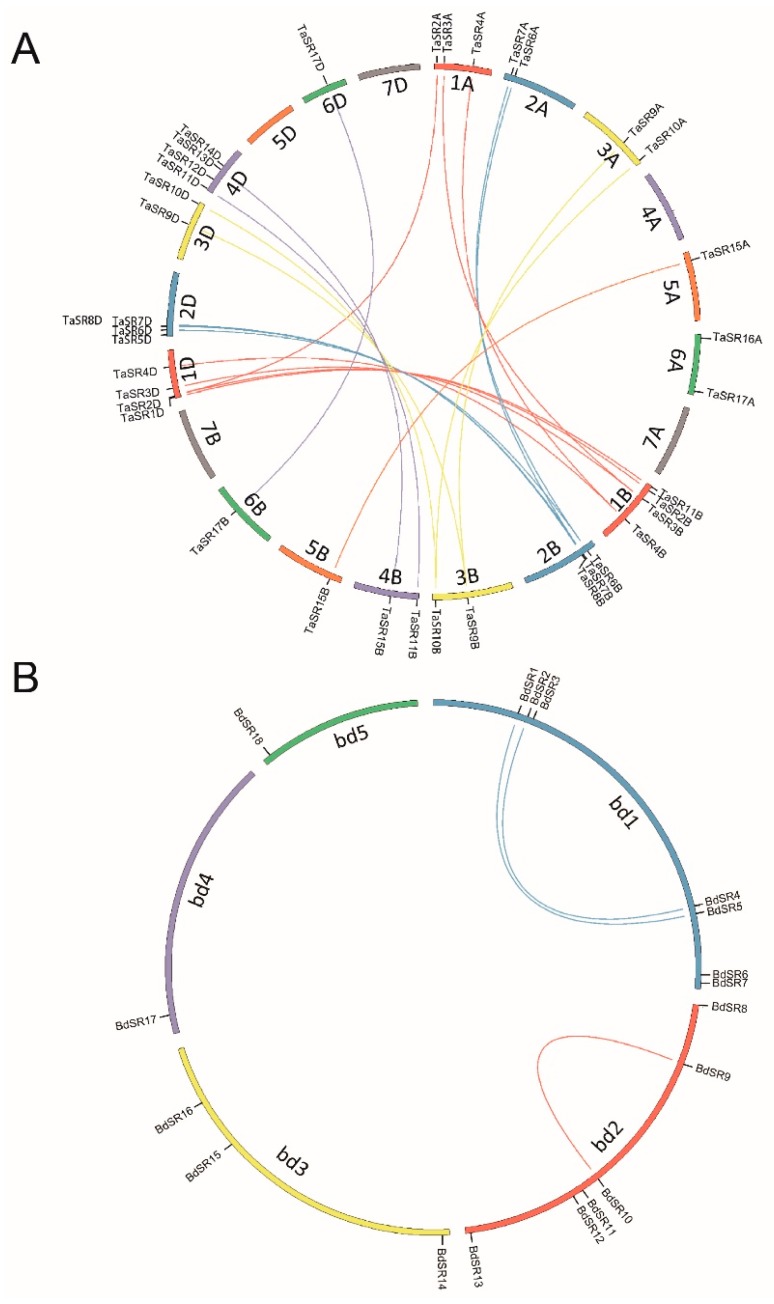
Schematic representations for the inter-chromosomal relationships of wheat and *B. distachyon* serine/arginine-rich (SR) protein genes. (**A**) Genomic locations and segment duplications of TaSR protein genes; (**B**) genomic locations and segment duplications of BdSR protein genes.

A total of 40 TaSR and 18 BdSR protein genes were identified, accounting for 0.040% and 0.034% annotated wheat and *B. distachyon* genes, respectively (Table S1). TaSR protein genes were named according to chromosome location and genomic homology (Figure 1A); and BdSR protein genes were named according to their distribution on chromosomes (Figure 1B). Except for BdSR11, all TaSR and BdSR protein genes were verified by ESTs (Expressed Sequence Tags) deposited in the NCBI database. Among 40 TaSR protein genes, 24 genes constitute 8 sets, and every set includes three homoeologous genes in A, B, and D sub-genomes respectively; 14 genes form 7 sets, and every set has two homoeologous genes.

The physical and chemical features of TaSR and BdSR proteins were also predicted. In wheat, the protein length varies from 205 (TaSR14, TaSR15) to 907 (TaSR27) amino acids; the PI varies from 9.43 (TaSR4, TaSR5) to 12.39 (TaSR24); the molecular weight is between 23.78 kDa (TaSR14) and 104.14 kDa (TaSR27). In *B. distachyon*, the protein length is from 205 (BdSR2) to 673 (BdSR14) amino acids; the PI varies from 9.65 (BdSR14) to 12.35 (BdSR13); the molecular weight is between 24.04 kDa (BdSR2) and 75.31 kDa (BdSR14). The grand average of hydropathicity (GRAVY) of all the SR proteins is negative and varies from −1.981 (TaSR12D) to −0.824 (TaSR9A) in wheat and from −1.64 (BdSR15) to −0.658 (BdSR14) in *B. distachyon*, representing a hydrophilic characteristic, probably because of the abundant arginine amino acids or absence of hydrophobic residues. The detailed information is listed in Tables S1 and S2.

### 2.2. Phylogenetic Tree of Plant SR Proteins

**Figure 2:**
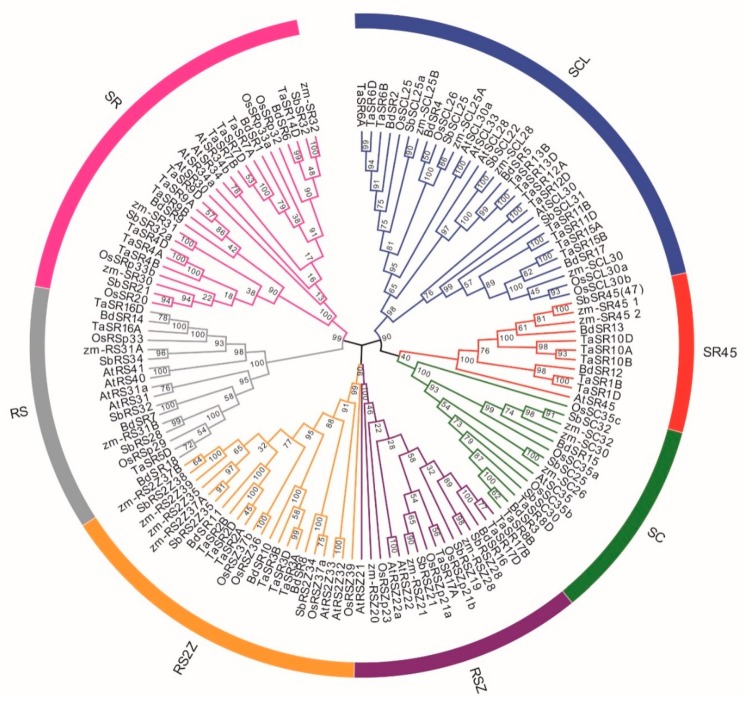
Phylogenetic tree of 138 plant SR proteins. The different colors indicate different subfamilies. Os, Sb, Zm, At, Bd, and Ta represent rice, *Sorghum bicolor*, *Z. mays*, *Arabidopsis*, *B. distachyon*, and wheat respectively.

**Figure 3:**
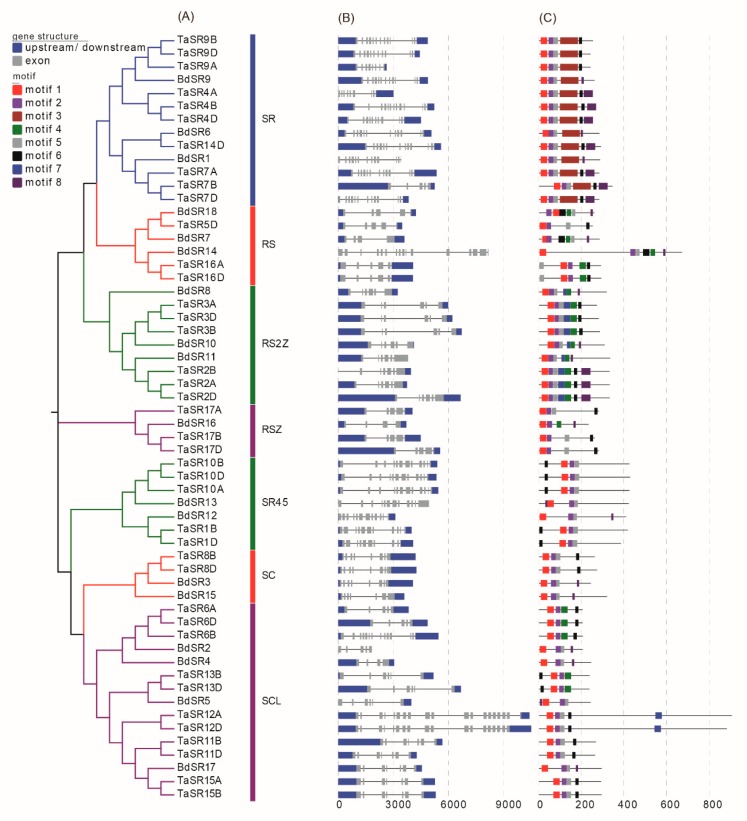
Phylogenetic relationships (**A**), motifs (**B**) and gene structure (**C**). The tree was constructed with 1000 bootstrap replications using MEGA7 based on the full-length protein sequence. The exon–intron structures of these genes were graphically displayed by the Gene Structure Display Server. The MEME Suite web server was used to predict the conserved motifs.

A neighbor-joining (NJ) phylogenetic tree was constructed based on the full-length alignment of SR proteins in wheat, *B. distachyon*, *Arabidopsis*, rice, maize, and sorghum (Figure 2 and Table S3). Consistent with previous reports, these SR proteins were classified into seven subfamilies; the SCL subfamily is the largest while SR45 subfamily is the smallest. There are 11 SCL, 10 SR, 6 RS2Z, 5 SR45, 3 RSZ, 3 RS, and 2 SC members in wheat; there are 4 SCL, 3 RS2Z, 3 SR, 3 RS, 2 SC, 2 SR45, and 1 RSZ members in *B. distachyon* (Figure 3A). About one half of TaSRs (20) and BdSRs (10) are plant-specific SRs (SCL, RS2Z, and RS subfamilies).

### 2.3. Gene Structure and the Conserved Motifs

Since gene structure is related to AS events, we analyzed the gene structures of TaSR and BdSR protein genes (Figure 3B). In general, all TaSR and BdSR protein genes have more than 3 exons. Generally, TaSR and BdSR protein genes in the same subfamily share a similar structure. For example, most RS2Z members have 6 exons, except for TaSR2B.

Additionally, we analyzed the motifs of TaSR and BdSR proteins. In total, eight types of motifs were identified (Figure 3C). Motifs 1 and 2 constitute the RRM1 domain, while motif 3, which constitutes the RRM2 domain, is only present in SR and RS members (Table S4). Motif 6 ([DG]Y[GX]R[RX][PR]SP) is one part of the RS domain, and motif 7 constitutes CCHC type zinc finger domain. Different from other SR proteins, the RS domain of SR45 members is in the N-terminus [25].

### 2.4. Synteny and Homologous Gene Pairs

**Figure 4:**
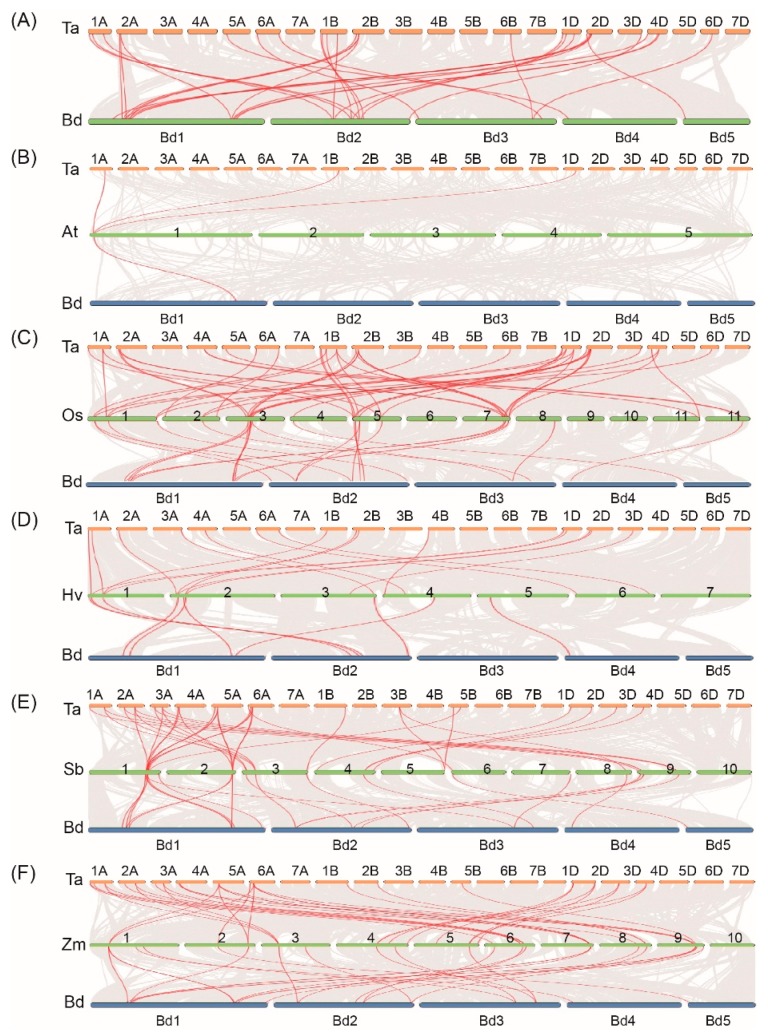
Syntenic relationships between SR protein genes in wheat (Ta), *B. distachyon* (Bd), *Arabidopsis* (At), rice (Os), *H.vulgare* (Hv), *S. bicolor* (Sb), and *Z. mays* (Zm). Gray lines indicate the collinear blocks within wheat/*B. distachyon* and other plant genomes, while the red lines highlight the syntenic *SR* proteins gene pairs. (**A**) Orthologous relationship of SR protein genes between wheat and *B. distachyon*. Orthologous relationship analysis of SR protein genes between (**B**) wheat/*B. distachyon* and *Arabidopsis*, (**C**) wheat/*B. distachyon* and rice, (**D**) wheat/*B. distachyon* and *H. vulgare*, (**E**) wheat/*B. distachyon* and *S. bicolor*, (**F**) wheat/*B. distachyon* and *Z. mays.*

To uncover the mechanism underlying the expansion of TaSR and BdSR protein genes, we investigated tandem and segmental duplications. As shown in Figure 4A, four and two segment duplication events were found in TaSR and BdSR protein genes, respectively (Table S5), while no tandem duplication event was found.

Orthologs and paralogs are two types of homologous sequences. Orthologs indicate genes in different species originated from a common ancestor, and orthologous genes may or may not have the same function. While paralogs indicate homologous genes within a species generated by gene duplications [26]. We investigated the orthologous of TaSR and BdSR protein genes. 22 homologous gene pairs were found between 22 TaSR and 12 BdSR protein genes (Figure 4A and Table S5). In addition, a total of 3, 46, 36, 36, 16 orthologous SR gene pairs between wheat and *Arabidopsis,* rice, barley, maize, sorghum were identified respectively (Figure 4B–F and Table S6); 1, 25, 25, 22, 9 orthologous SR gene pairs between *B. distachyon* and *Arabidopsis,* rice, barley, maize, sorghum were identified respectively (Figure 4B–F and Table S7).

### 2.5. Cis-Acting Regulatory Elements 

*Cis*-elements in the promoters affect the expression of genes, so TaSR and BdSR protein gene promoters were investigated by using the new PLACE (Plant cis-acting regulatory DNA elements) program [27]. As shown in Table S8 and Table S9, 92 *cis*-elements were found both in TaSR and BdSR protein genes and they can be divided into three types: development-related, hormone-responsive, and abiotic/biotic stress responsive. *Cis*-elements related to development including light responsivity (CACTFTPPCA1, CAATBOX1, EBOXBNNAPA, ARR1AT, GTGANTG10), endosperm expression (DOFCOREZM), and pollen expression (GTGANTG10); *cis*-element related to abiotic stresses include cold response (LTRECOREATCOR15), heat response (CCAATBOX1), and drought responsivity (MYCCONSENSUSAT, MYB2CONSENSUSAT); and *cis*-element related to hormone responsivity include ABA (DPBFCOREDCDC3, RYREPEATBNNAPA, MYCATRD22), SA (ASF1MOTIFCAMV), and so on. Among these 92 *cis*-elements, DOFCOREZM, CACTFTPPCA1, CAATBOX1, MYCCONSENSUSAT, ARR1AT, GTGANTG10, POLLEN1LELAT52, MYCCONSENSUSAT, and WRKY71OS were identified in the promoters of all TaSR and BdSR protein genes. Combined with the phylogenetic tree, results showed that the phylogenetically similar genes shared identical *cis*-elements (Table S10).

### 2.6. Predicted Protein Interactions

**Figure 5:**
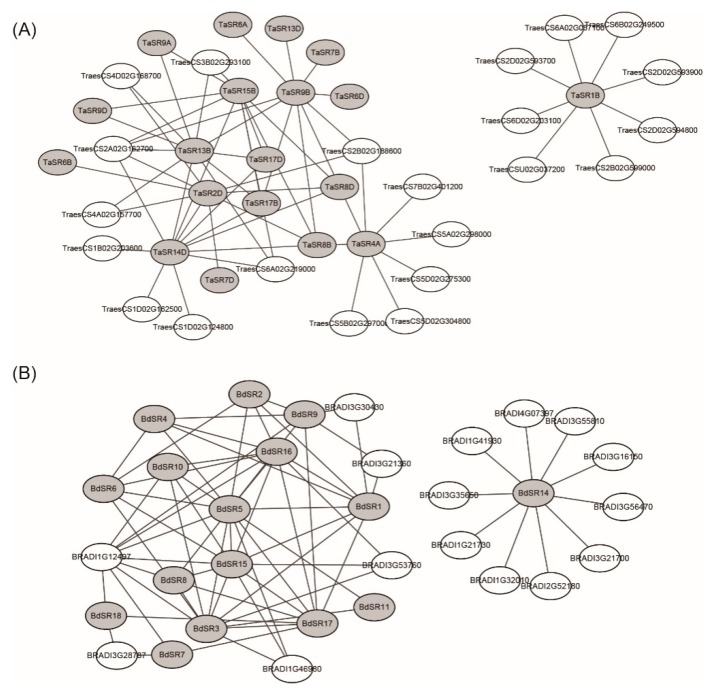
Protein–protein interaction network of (**A**) TaSR and (**B**) BdSR proteins. The gray elliptical represent the TaSR or BdSR proteins, the white elliptical represent the other wheat or *B. distachyon* proteins, and the black lines represent the interaction relationship between proteins.

As components of the spliceosome, SR proteins interact with other proteins and form complexes. Several plant SR proteins have been shown to interact with other SR members or other spliceosomal proteins [28]. For example, SR45 could interact with U1-70K, U2AF^35^a, and U2AF^35^b [29]. To further understand the functional mechanism of TaSR and BdSR proteins, interaction networks of TaSR or BdSR proteins were built (Figure 5). In wheat, 69 protein pairs were predicted with high confidence (score > 0.900) (Table S11). Among them, 31 are interactions between different SR members, and 38 are interactions between 19 TaSR proteins and 22 non-SR proteins. In *B. distachyon*, 75 protein pairs were predicted with high confidence between 16 SR proteins and 16 non-SR proteins (Table S12). Among those 16 *B. distachyon* proteins, 6 have one or two RRM domains. Most TaSR and BdSR proteins involved in protein interactions belong to SR and SCL subfamilies.

**Figure 6:**
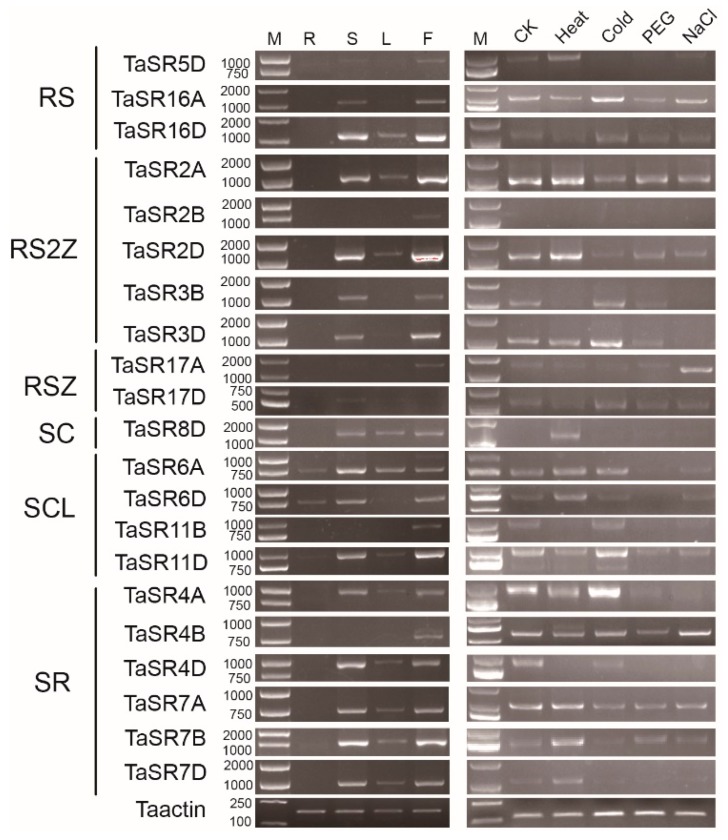
Expression pattern of TaSR protein genes. M, R, S, L, and F indicate DL2000 marker, root, stem, leaf, and inflorescences in plants. CK (H_2_O), Heat, Cold, PEG, and NaCl represent different abiotic stresses.

**Figure 7:**
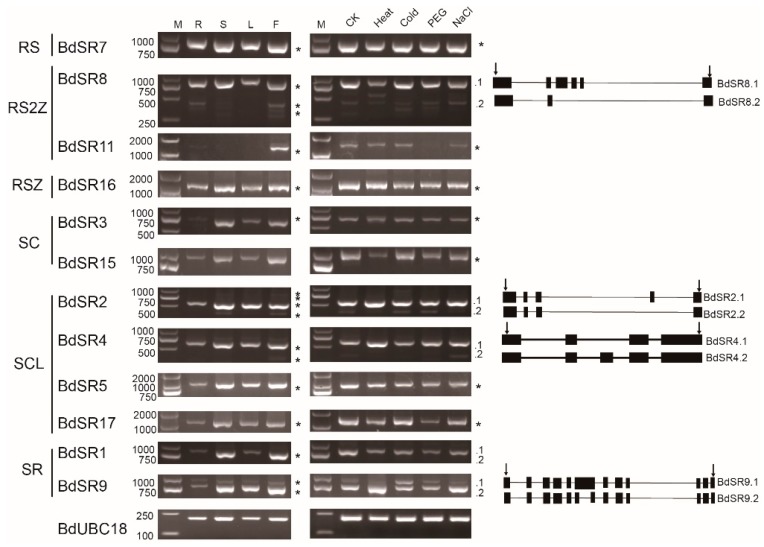
Expression pattern and AS pattern of BdSR protein genes. M, R, S, L, and F indicate DL2000 marker, root, stem, leaf, and inflorescences in plants. CK (H_2_O), Heat, Cold, PEG, and NaCl represent different abiotic stresses. The diagram on the right of the polymerase chain reaction (PCR) indicate different isoforms, arrows indicate primers.

Since most of these SR protein genes encoding interacting proteins have overlapping expression patterns (Figure 6 and Figure 7), we analyzed the expression patterns of non-SR protein genes involved in protein–protein interaction networks (Figure S2). Compared with results in Figure 6 or Figure 7, most of those non-SR protein genes have overlapped expression patterns with their partners.

### 2.7. Expression and Alternative Splicing Patterns

To better understand the functions of SR proteins, we performed a semi-quantitative polymerase chain reaction (PCR) to analyze the expression patterns and splicing patterns of TaSR and BdSR protein genes. Among 23 selected TaSR protein genes, except for *TaSR1B* and *TaSR1D*, 21 genes are expressed (Figure 6). Most of them are highly expressed in stems and inflorescences, and two genes are expressed in roots. Homoeologous genes *TaSR7A*, *TaSR7B*, and *TaSR7D* display similar expression patterns. They are expressed in stems and inflorescences at a high level, and in leaves at a low level. Among *TaSR4A*, *TaSR4B*, and *TaSR4D*, another set of homoeologous genes, *TaSR4B* is only expressed in inflorescences, while *TaSR4A* and *TaSR4D* are expressed in stems, leaves, and inflorescences. Similarly, *TaSR2B* displayed different expression pattern, compared to *TaSR2A* and *TaSR2D*. Under heat, cold, drought, and salt stresses, the expression of these 21 genes do not display regular expression patterns in seedlings.

For 18 BdSR protein genes (Figure 7), the expression of 6 genes could not be detected with two pair of primers, probably because of extremely low expression level; 11 expressed genes displayed extensive expression pattern, and they are expressed in roots, stems, leaves, and inflorescences, although the expression level in roots is low (Figure 7); another gene, *BdSR11*, is only expressed in inflorescences. Under abiotic stresses, most of these genes are constitutively expressed (Figure 7). In general, TaSR and BdSR protein genes are highly expressed in inflorescences, suggesting important roles at reproductive stage.

In *B. distachyon*, *BdSR4* (*SCL33*), *BdSR9* and *BdSR11* are alternatively spliced [30]. In addition to *BdSR9* and *BdSR11*, we found AS occurred in *BdSR4* and *BdSR15* by analyzing ESTs (Figure S1) [31]. By analyzing the RT-PCR results, we found that *BdSR2*, *BdSR4*, *BdSR8*, and *BdSR9* are probable alternative spliced genes. By DNA sequencing, we verified these AS events and acquired their AS pattern. Taken together, 6 genes (*BdSR2*, *BdSR4*, *BdSR8*, *BdSR9*, *BdSR11*, and *BdSR15*) are alternatively spliced, accounting for 1/3. Compared to *Arabidopsis*, rice, maize, and sorghum, the number of alternative spliced genes and the number of transcripts is much less. More surprisingly, as shown in Figure 6, no AS event was found in 21 expressed TaSR protein genes.

## 3. Discussion

### 3.1. Conservation between Wheat and B. distachyon SR Protein Genes

In this study, 40 TaSR and 18 BdSR protein genes were identified, accounting for 0.040% and 0.034% annotated wheat and *B. distachyon* genes, similar to that of rice (0.038%) and *Sorghum bicolor* (0.042%) [32]. They are classified into seven subfamilies, and the proportion of plant-specific subfamily members in TaSR (50%, 20/40) and BdSR (55.56%, 10/18) were similar. 

Additionally, gene structures and protein motifs of TaSRs and BdSRs are similar within the same subfamilies. In the promoters of these SR protein genes, 92 *cis*-elements were found both in TaSR and BdSR protein genes, and they associate with plant growth/development and abiotic stress response. Among these 92 *cis*-elements, AAAG, YACT, CAAT, CANNTG, NGATT, GTGA, CANNTG, and TGAC occurred at a high proportion in all TaSR and BdSR protein genes. The phylogenetically similar genes shared identical *cis*-elements (Table S10). Furthermore, several SRs in plants have paralogs due to whole-genome and segmental duplications, a common feature in plants [11,33]. In this study, 4 TaSR and 2 BdSR protein genes were found to have undergone segmental duplication. Interestingly, no tandem duplication of TaSR and BdSR protein genes was detected, consistent with the report in *Brassica rapa* [34]. Moreover, 22 homologous gene pairs were found between 22 TaSR and 12 BdSR protein genes, and most TaSR and BdSR protein genes were highly expressed in inflorescences. Taken together, it is possible to conclude that TaSR and BdSR protein genes are conserved in some respects.

### 3.2. Plant SR Proteins May Function in Plant Development and in Response to Environmental Stresses

In *Arabidopsis*, different expression patterns of SR protein genes suggest their extensive functions [6,14,35]. For example, overexpression of *Arabidopsis SRp30*, *RSZ33*, or mutations of *SC35* and *SCL* genes cause abnormal plant development [13,14,15]. Two transcripts of *Arabidopsis* SR45 function differently in a tissue-specific manner: isoform SR45.1 functions in flower development while isoform SR45.2 functions in root growth [18].

Additionally, the AS of plant SR protein genes is also dramatically altered under various stresses, implying their functions in response to environmental stresses. In *Arabidopsis* and rice, the alternative splicing pattern of most SR protein genes showed remarkable change under various abiotic stresses, including temperature stress [7,17,36], high salinity [37], and high light irradiation [21].

Although the functions of wheat and *B. distachyon* SR protein genes have not been reported, most of them are extensively expressed, especially at high level in the inflorescences, indicating TaSR and BdSR proteins might regulate the plant growth and development. Meanwhile, many development-related, hormone-responsive, and abiotic/biotic stress responsive *cis*-elements were identified in the promoters of *TaSR* and *BdSR* protein genes, further indicating their functions in development and in response to environmental stresses. 

In *B. distachyon*, multiple intron-retaining splice variants of *SCL33* (*BdSR4* in this study) was identified during virus infection, indicating that it modulates the response to biotic stresses [30]. In this study, two transcripts of *BdSR9* were found under heat and cold stresses, also indicating its function in response to abiotic stresses.

## 4. Materials and Methods

### 4.1. Identification of SRs in Plants

The wheat and *B. distachyon* genome sequences, protein sequences, coding sequences, and upstream 1.5-kb genomic DNA sequences were downloaded from Ensembl plants [38] and Phytozome V12 [39] respectively. The SR protein sequences of *Arabidopsis*, rice, maize, sorghum, and barley used in this study were downloaded in the Ensembl plants database [38]. 

To identify the SRs in wheat and *B. distachyon*, 18 *Arabidopsis* and 22 rice SR protein sequences were selected to build a hidden Markov model (HMM), then searched against the wheat and *B. distachyon* genome protein sequences using a threshold of *e*-value <1e-5 and the length of amino acid ≥200 aa. Subsequently, manual corrections were performed to remove the alternative splicing events and any redundancy. Finally, the NCBI-CDD (NCBI Conserved Domains Database) and SMART (Simple Modular Architecture Research Tool) were performed to confirm whether the obtained SR proteins had conserved RRM and RS domain. 

The theoretical isoelectric point (PI), molecular weight (MW), grand average of hydropathicity (GRAVY) were predicted by the ProtParam tool [40]. The subcellular location was predicted by the CELLO v2.5 [41]. To further evaluate the existence of TaSR and BdSR protein genes, the coding domain sequences (CDS) were used to search against the wheat and *B. distachyon* expressed sequence tag (EST) database using the BLASTN tool in the NCBI database. To construct the alternative splicing graph of potential splice variants of BdSR protein genes, the ESTs of the *B. distachyon* were downloaded from dbEST [42] and then aligned to the corresponding genomic contigs and genome sequences using the previous method by Kianianmomeni A, Cheng SO, Rätsch G and Hallmann A [31].

### 4.2. Multiple Alignments and Phylogenetic Analyses

The neighbor-joining (NJ) phylogenetic tree was constructed by using MEGA-X software [43] with 1000 bootstrap replications and Jones-Taylor-Thornton model based on full-length protein sequence alignments using the T-COFFEE, a web server for the multiple sequence alignment of protein sequence [44]. The phylogenetic trees were visualized by Evolview online [45].

### 4.3. Cis-Acting Regulatory Elements, Gene Structure, and Conserved Motifs Analyses

The *cis*-elements in the 1.5-kb upstream genomic DNA sequences of wheat and *B. distachyon* were submitted to New PLACE database, a database of plant *cis*-acting regulatory DNA elements, to predict the *cis*-acting elements [27]. Gene structures of SR protein genes were deduced using the Gene Structure Display Server (GSDS) online [46]by using the CDS and genome sequence of TaSR and BdSR protein genes. The conserved motifs were investigated by using the MEME Suite web server with the maximum number of motif sets at 8 and the optimal width of motifs from 5 to 200 amino acids [47].

### 4.4. Chromosome Location and Gene Duplication Analyses

Chromosome distribution information of TaSR and BdSR protein genes was obtained from the Ensembl plants and Phytozome databases, respectively. Gene duplication analysis including tandem and segmental duplication in wheat and *B. distachyon* as well as the synteny relationship analyses of wheat, *B. distachyon*, and other plant species were predicted by the MCScanX program [48] and visualized by Dual Systeny Plotter software by CJ-Chen [49]. Genomic locations and segment duplication were visualized by using the Circos v0.55 [50].

### 4.5. Prediction of Protein–Protein Interactions

The interactions between SRs and other proteins were predicted using the STRING v11 online program [51] and visualized by the Cytoscape v3.4.0 software program [52]. To predict the interaction network of TaSR or BdSR proteins on the basis of *Arabidopsis* proteins, the homologs of these interaction proteins in wheat and *B. distachyon* were identified using the best hits of BLASTP analysis with a threshold of *e*-value <1e-5, then were predicted by the STRING v11. 

### 4.6. Plant Growth, Stress Treatment, and Semi Quantitative Real-Time Polymerase Chain Reaction (RT-PCR) Analyses

The cultivar of *T. aestivum* Chinese Spring and *B. distachyon* Bd-21 were planted in an artificial climate chamber at 26/22 °C (day/night) with a photoperiod of 16/8 h (day/night). For tissues analysis, root, stems, leaves, and inflorescences were collected at the heading stage. For different abiotic stresses, 2-week-old whole seedling plants under hydroponics cultivation and were collected after being subjected to H_2_O(CK), heat (42 °C), cold (4 °C), drought (20% PEG2000), and salt (200 mM NaCl) for 2 h. The methods of RNA extraction and cDNA synthesis were carried out as described in our previous study [36]. For validation of alternative splicing in SR protein genes, primers were designed by Primer Primer5 software for semi-quantitative RT-PCR, wheat actin gene and *B. distachyon* UBC18 gene were used as control as reported in our previous study [36,53]. The following program was used for RT-PCR: 94 °C for 5 min followed by 32 or 40 cycles (32 cycles for the actin/UBC18 gene, and 40 cycles for the SR protein genes) at 94 °C for 30 s, 55–60 °C for 30 s, and 72 °C for 40–60 s, followed by 72 °C for 5 min. The denaturation and annealing temperature and time were based on the sequence’s length of targeted genes and primers. The primers are listed in Table S13.

## 5. Conclusions

SR proteins are essential splicing factors for constitutive splicing and are highly conserved in plants and metazoans. We undertook a comprehensive genome-wide characterization and expression analysis of the SR protein family in wheat and *B. distachyon*. A total of 40 *TaSR* and 18 *BdSR* genes were identified and classified into seven subfamilies. Proteins within the same subgroup shared similar gene structures and protein motifs. Synteny analysis showed that segmental duplications contributed to the expansion of *SR* genes in wheat and *B. distachyon*. Moreover, SR proteins might have distinct biological functions by protein–protein interactions and involvement in plant development. This study provides strong evidence that SR proteins play an important role in alternative splicing and plant development.

## Supplementary Materials

The following are available online at https://www.mdpi.com/2223-7747/8/7/188/s1: Figure S1: EST validation of four BdSR protein genes, Figure S2. Expression pattern of the co-expression genes. M, R, S, L, and F indicate DL2000 marker, root, stem, leaf, and inflorescences in plants, Table S1: Table S1: The detailed information of TaSRs, Table S2: The detailed information of BdSRs, Table S3: The SR proteins in other plants, Table S4: The identified conserved domains in SR proteins, Table S5: Wheat and *B. distachyon SR* gene duplication events, Table S6: The homologous genes between wheat and other plant genes, Table S6: The homologous genes between *B. distachyon* and other plant genes, Table S8: Numbers of known stress-related elements in the promoter regions of *TaSR* genes, Table S9: Numbers of known stress-related elements in the promoter regions of *BdSR* genes, Table S10: Three type *cis*-elements in the promoter regions of *TaSR* and *BdSR* genes, Table S11: The prediction of protein-protein interaction pairs in wheat SR proteins, Table S12: The prediction of protein-protein interaction pairs in *B. distachyon* SR proteins, Table S13: Primer used in this study.
